# *Enterobacter cloacae* administration induces hepatic damage and subcutaneous fat accumulation in high-fat diet fed mice

**DOI:** 10.1371/journal.pone.0198262

**Published:** 2018-05-30

**Authors:** Anniina Keskitalo, Eveliina Munukka, Raine Toivonen, Maija Hollmén, Heikki Kainulainen, Pentti Huovinen, Sirpa Jalkanen, Satu Pekkala

**Affiliations:** 1 Institute of Biomedicine, University of Turku, Turku, Finland; 2 Department of Clinical Microbiology, Turku University Hospital, Turku, Finland; 3 Medicity Research Laboratory, University of Turku, Turku, Finland; 4 Faculty of Sport and Health Sciences, University of Jyväskylä, Jyväskylä, Finland; East Tennessee State University, UNITED STATES

## Abstract

Accumulating evidence indicates that gut microbiota plays a significant role in obesity, insulin resistance and associated liver disorders. Family *Enterobacteriaceae* and especially *Enterobacter cloacae* strain B29 have been previously linked to obesity and hepatic damage. The underlying mechanisms, however, remain unclear. Therefore, we comprehensively examined the effects of *E*. *cloacae* subsp. *cloacae* (ATCC® 13047™) administration on host metabolism of mice fed with high-fat diet (HFD). C57BL/6N mice were randomly divided into HFD control, chow control, and *E*. *cloacae* treatment groups. The *E*. *cloacae* treatment group received live bacterial cells in PBS intragastrically twice a week, every other week for 13 weeks. Both control groups received PBS intragastrically. After the 13-week treatment period, the mice were sacrificed for gene and protein expression and functional analyses. Our results show that *E*. *cloacae* administration increased subcutaneous fat mass and the relative proportion of hypertrophic adipocytes. Both subcutaneous and visceral fat had signs of decreased insulin signaling and elevated lipolysis that was reflected in higher serum glycerol levels. In addition, *E*. *cloacae -*treated mice had significantly higher hepatic AST and AST/ALT ratio, and their liver histology indicated fibrosis, demonstrating that *E*. *cloacae* subsp. *cloacae* administration promotes hepatic damage in HFD fed mice.

## Introduction

According to WHO [1], the prevalence of obesity has almost tripled during the past four decades. Consequently, the frequency of obesity-associated metabolic disorders, including non-alcoholic fatty liver disease (NAFLD), has exacerbated. The prevalence of obesity and metabolic disorders is considered to increase due to changes in environmental factors such as diet and lifestyle, and accumulating evidence is indicating that gut microbiota plays a role in the development of these diseases [2]. Gut microbes have an important role in host metabolism, as they, for instance, ferment otherwise indigestible dietary polysaccharides into short-chain fatty acids, promote the absorption of monosaccharides, affect the activity of certain gut enzymes and increase anabolic metabolism [3,4]. In the past decades, several studies have reported associations between gut microbiota composition and obesity; obese individuals seem to have reduced gut microbiota diversity compared to lean individuals (for review, see [5]), and the abundance of specific microbial taxa, functional genes, as well as metabolic activities differ significantly between the obese and lean individuals [6,7]. In addition, gut microbes have been shown to participate in the regulation of host lipogenesis and fat storage [3,8].

Recently, the microbial surface molecules have been suggested to induce obesity-linked inflammatory and metabolic effects on the host [9]. For instance, lipopolysaccharide (LPS), the major component of the outer membrane of Gram-negative bacteria, has been shown to cause inflammation when entering the circulation [10,11]. High-fat diet (HFD) related increased energy intake has been shown to increase gut permeability in mice, leading to chronic elevation of circulating LPS. This state, called metabolic endotoxemia, has been further linked to elevated body weight, liver and adipose tissue weight, adipose tissue and liver inflammation, as well as fasted hyperglycemia and insulinemia [10,12]. Thus, it seems possible that certain LPS-possessing gut microbes might contribute to metabolic endotoxemia and play a role in the development of obesity and metabolic diseases [12]. Interestingly, LPS from *Enterobacteriaceae* has been reported to exhibit a significantly higher endotoxin activity than the LPS from more abundant gut bacteria like *Bacteroidetes* [13]. One of these endotoxin-possessing *Enterobacteriaceae* species associated with obesity and liver damage is *Enterobacter cloacae (E*. *cloacae*; [14]). This facultative anaerobic, Gram-negative, flagellated bacterium is a member of normal human gut microbiota, but can also cause infections like bacteremia, urinary tract infections, and intra-abdominal infections [15,16]. Interestingly, we have recently shown that flagellin, a structural protein of bacterial locomotive organelle flagellum, may contribute to obesity and hepatic fat content by affecting adipose tissue [17,18].

An increased abundance of *E*. *cloacae* in humans was initially linked to obesity in a case study by Zhao et al., where the reduction of intestinal *E*. *cloacae* strain B29, from 35% to non-detectable levels, was associated with a parallel reduction in endotoxin load and a significant weight loss in a patient with morbid obesity [14]. Furthermore, the same bacterial strain, isolated from this patient, initiated obesity and triggered inflammation when introduced to HFD germ-free mice [14,19]. The above-mentioned studies suggest that *E*. *cloacae* may contribute to obesity, possibly through an endotoxin or flagella-induced inflammation-mediated mechanism. In this study, we explored the effects of *E*. *cloacae* administration on HFD female mice. Female mice were studied, because even though longitudinal studies report higher incidence of NAFLD in males, cross-sectional studies suggest that females may be more prone to develop non-alcoholic steatohepatitis (NASH; for review, see [20]). *E*. *cloacae* subsp. *cloacae* (ATCC® 13047™) was used in the experiments, as it is the type strain of *E*. *cloacae* with a fully sequenced genome, and phylogenetically a close relative to *E*. *cloacae* strain B29 [14].

## Materials and methods

### In vitro cultures

*E*. *cloacae* subspecies *cloacae* ATCC® 13047™ were maintained at +37°C on Fastidious anaerobe blood agar plates (Lab M Limited, Lancashire, UK). Intragastric inoculums were prepared by suspending the bacterial cells in phosphate-buffered saline (PBS) at an approximated, turbidity-based cell density of 9 × 10^8^ CFU/ml. The volume of one inoculum, including approximately 2 × 10^8^ bacterial cells, was 220 μl. At the time of transferring the suspension to the syringe, the viability of the suspended bacterial cells was confirmed by plating an aliquot of the suspension on Fastidious anaerobe blood agar plate. After that, the syringe was sealed and transported to the animal facility.

### Animals

The experiment was approved by the national ethics committee of animal experimentation in Finland (ESAVI/7258/04.10.07/2014), and all work was performed in strict accordance with the guidelines of the Act on the Use of Animals for Experimental Purposes by the Ministry of Agriculture and Forestry, Finland. Eight-week-old C57BL/6N female mice (Charles River, Europe) were randomly divided into HFD control, chow control, and *E*. *cloacae* treatment groups (n = 6/group) and housed in IVC racks under SPF conditions. The mice received food and water *ad libitum* and were maintained on a 12/12-hour light/dark cycle. The irradiated HFD (58126 DIO Rodent Purified Diet w/60% energy) and the matching irradiated chow diet (58124 DIO Rodent Purified Diet w/10% energy) were purchased from Labdiet/Testdiet, UK. *E*. *cloacae*-treatment group received approximately 2 × 10^8^ *E*. *cloacae* cells in PBS intragastrically twice a week, every two weeks for 13 weeks. Both control groups received sterile PBS. Body weight was measured weekly at the same time of day. Food consumption was monitored by weighing the consumed food per cage in five 24 h periods (weeks 1, 3, 6, 9, and 12). Average food intake per mice was calculated by dividing the total food consumption of the cage by the number of mice residing in the cage. The approximate daily energy intake of the mice was calculated based on the composition of the diets (Table 1). One mouse from the HFD/control group and one mouse from the *E*. *cloacae* treatment group had to be excluded from the study due to significant weight loss during the treatment period.

**Table 1:** Composition of the diets.

	Chow diet^a^		High-fat diet^b^	
Ingredient	%	% kcal	%	% kcal
Protein	16.9	18.0	23.1	18.1
Carbohydrates	67.4	71.8	25.9	20.3
Fat	4.3	10.2	34.9	61.6
Fiber	4.7		6.5	
kcal/g (kJ/g)	3.76 (15.7)		5.10 (21.3)	

^a^TestDiet® DIO Rodent Purified Diet w/10% Energy from Fat

^b^TestDiet® DIO Rodent Purified Diet w/60% Energy from Fat

### Tissue collections and blood analyses

After the 13-week treatment period, the overnight-fasted mice were euthanized by CO_2_ exposure, and whole blood was drawn without delay by cardiac puncture. Finally, the euthanasia was confirmed by cervical dislocation. Serum glycerol, AST, and ALT were analysed using KONELAB 20XTi analyser (Diagnostic Products Corporation, Los Angeles, CA, USA). The subcutaneous (SAT) and visceral adipose tissue (VAT), intestines and liver were harvested, weighed with an electronic scale, immersed in liquid nitrogen and stored at -80°C. For the subsequent analyses of gene expression, protein phosphorylation, and fat content, the tissues were pulverized in liquid nitrogen.

### Liver triglyceride measurement

Total lipids were extracted from ~20 mg liver tissue as previously described [21]. The dried lipid extract was dissolved in ethanol and the triglyceride content was determined with KONELAB 20XTi analyser.

### Histological and immunohistochemical analyses

Frozen liver sections were stained with anti-smooth muscle actin (SMA) and hematoxylin & eosin (H&E), and adipose tissues with anti-mouse CD45 (clone 30F11, BD 553076) and H&E as previously described [21]. The adipocyte sizes from 500 randomly selected cells in each sample were determined with CellProfiler 2.2.0. The number of CD45 positive cells was calculated manually from the stained tissues. On average, cells from ~10 fields of view were calculated, and the results are presented as the number of cells per field.

### Gene expression and protein phosphorylation analyses

Real-time quantitative PCR and Western blot were performed as previously described [21,22]. Total RNA was extracted from pulverized tissues using TissueLyser (Qiagen, Hilden, Germany) and Trizol reagent (Invitrogen, Carlsbad, CA, USA), according to the supplier’s protocol. Total RNA was reversely transcribed according to the manufacturer’s instructions using High Capacity cDNA Synthesis Kit (Applied Biosystems, Foster City, CA, USA), and real-time PCR analyses were performed according to MIQE guidelines using in-house designed primers (purchased from Invitrogen), iQ SYBR Supermix and CFX96™ Real-time PCR Detection System (Bio-Rad Laboratories, Richmond, CA, USA). Each sample was analyzed in duplicate and PCR cycle parameters were as follows: +95°C for 10 min, 40 cycles at +95°C for 10 s, at +60°C for 30 s and at +72°C for 30 s, followed finally by 5 s at +65°C. The relative expression values were calculated against standard curve with the CFX96™ Software and normalized to the expression level of *ACTB* mRNA. The sequences of the qPCR primers are presented in S1 Table. Western blots were done with primary antibodies from Cell Signaling Technology (Danvers, MA, USA). The blots were scanned with Odyssey CLX Infrared Imager (Li-COR Biosciences, Lincoln, NE, USA), using Odyssey anti-rabbit IRDye 800CW and anti-mouse IRDye 680RD as secondary antibodies.

### Statistics

Statistical analyses were performed with either SPSS Statistics 22 or JMP Pro 12. All data were checked for normality using the Shapiro-Wilk´s test. For body weight, one-way ANOVA and Tukey’s range test were used to analyze the differences between the study groups. As most of the other data were not normally distributed, the group differences in gene expression, protein phosphorylation levels, liver triglyceride content, and in the numbers of leukocytes were analyzed by non-parametric Kruskal-Wallis test and Mann Whitney U test.

## Results

### *E*. *cloacae* administration increases fat mass and the percentage of hypertrophic adipocytes, reduces insulin receptor β expression and increases lipolysis in subcutaneous adipose tissue

**Fig 1:**
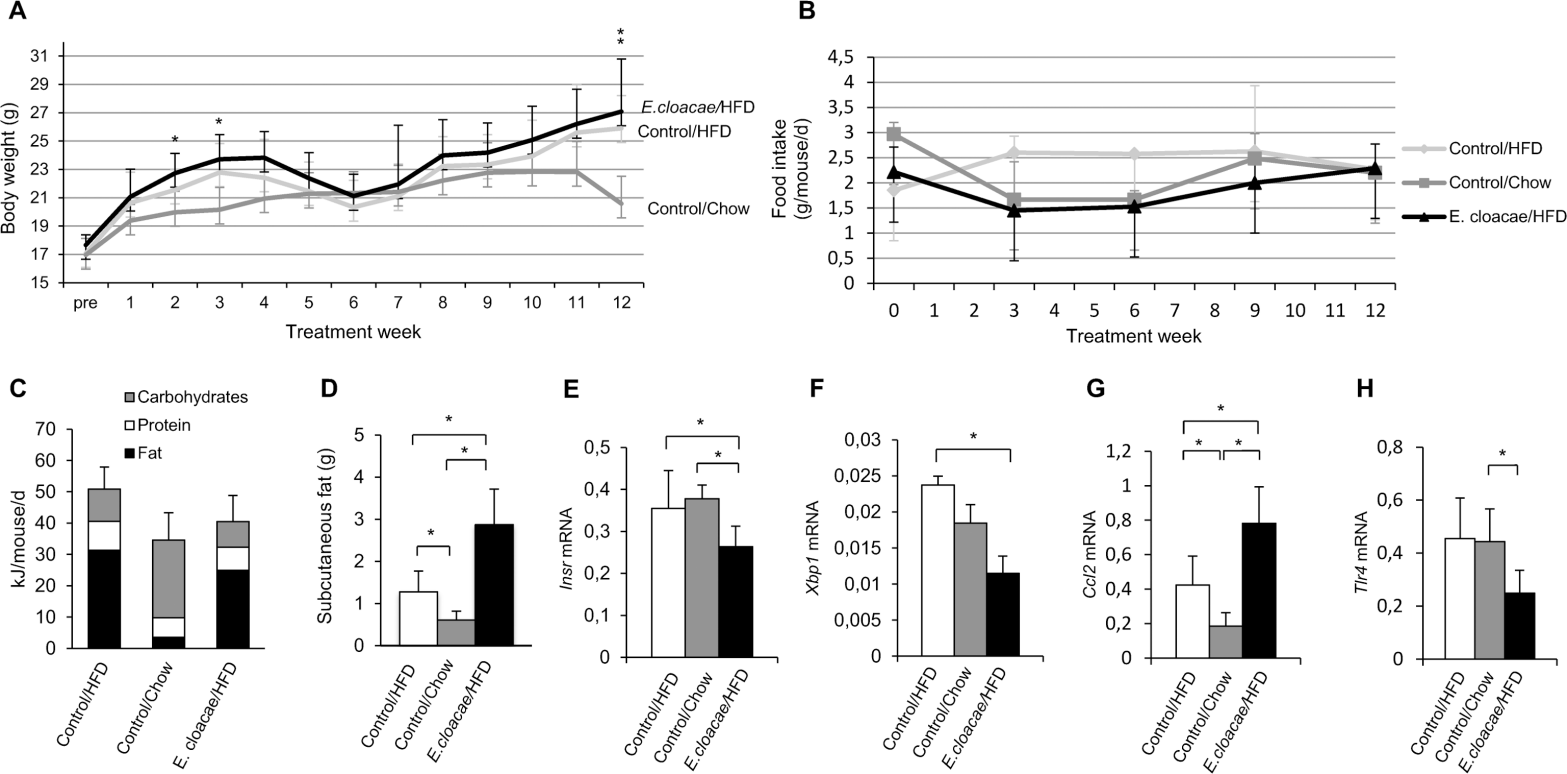
*E*. *cloacae* administration increases subcutaneous adipose tissue mass and reduces insulin receptor β expression in SAT. (A) Weight gain during the treatment period. (B) Food consumption during the treatment period. (C) Average daily energy intake during the treatment period. At necropsy: (D) SAT mass; (E) *Insr* expression; (F) *Xbp1* expression; (G) *Ccl* expression; (H) *Tlr4* expression. All data are presented as mean ± SD. n was 4-6/group when statistical outliers were excluded. The statistical significance was set to p<0.05 and the significant differences are presented with lines and * between the groups.

The weight gain during the experiment is shown in Fig 1A. In the second and third week of the study, the *E*. *cloacae* -treated mice differed in weight from the chow controls (p = 0.019 & 0.015, respectively; Fig 1A). At the endpoint of the study, the chow controls weighted significantly less than the *E*. *cloacae* -treated mice and the HFD controls (p = 0.004 and p = 0.014, respectively). No statistically significant differences in the average food consumption or in the approximate energy intake were observed between the study groups (Fig 1B and 1C). Visual observation of the graphics, however, suggests that the HFD control mice might have consumed more food than the chow controls and the *E*. *cloacae* -treated mice.

Compared to the HFD control, the chow controls had lower (p = 0.009, Fig 1D) and the *E*. *cloacae* treatment group higher (p = 0.016, Fig 1D) SAT mass. The *E*. *cloacae* treatment group expressed less insulin receptor β (*Insr*) than the HFD (p = 0.008, Fig 1E) and chow controls (p = 0.004, Fig 1E) in the SAT, which is a possible indicator of declined insulin sensitivity. The increased fat mass has been previously shown to associate with endoplasmic reticulum (ER) stress and infiltrations of leukocytes into the adipose tissue, which subsequently exacerbates insulin resistance [23]. *E*. *cloacae* treatment group, however, expressed less ER stress-related spliced X-box binding protein 1 (XBP-1) encoding gene (*Xbp1*) than the HFD controls (p = 0.047, Fig 1F). On the other hand, the expression of monocyte chemoattractant protein-1 (MCP-1) encoding gene (*Ccl2*) was elevated in the *E*. *cloacae* treatment group and the HFD control group compared to the chow controls (p = 0.004 and p = 0.009, respectively; Fig 1G). Further, the *Ccl2* expression was higher in the *E*. *cloacae* treatment group than in the HFD controls (p = 0.032; Fig 1G). Surprisingly, however, the expression of LPS-recognizing *Tlr4* was significantly lower in the *E*. *cloacae* -treated mice than in the chow controls and the HFD controls (p = 0.009 and p = 0.032, respectively; Fig 1H).

**Fig 2:**
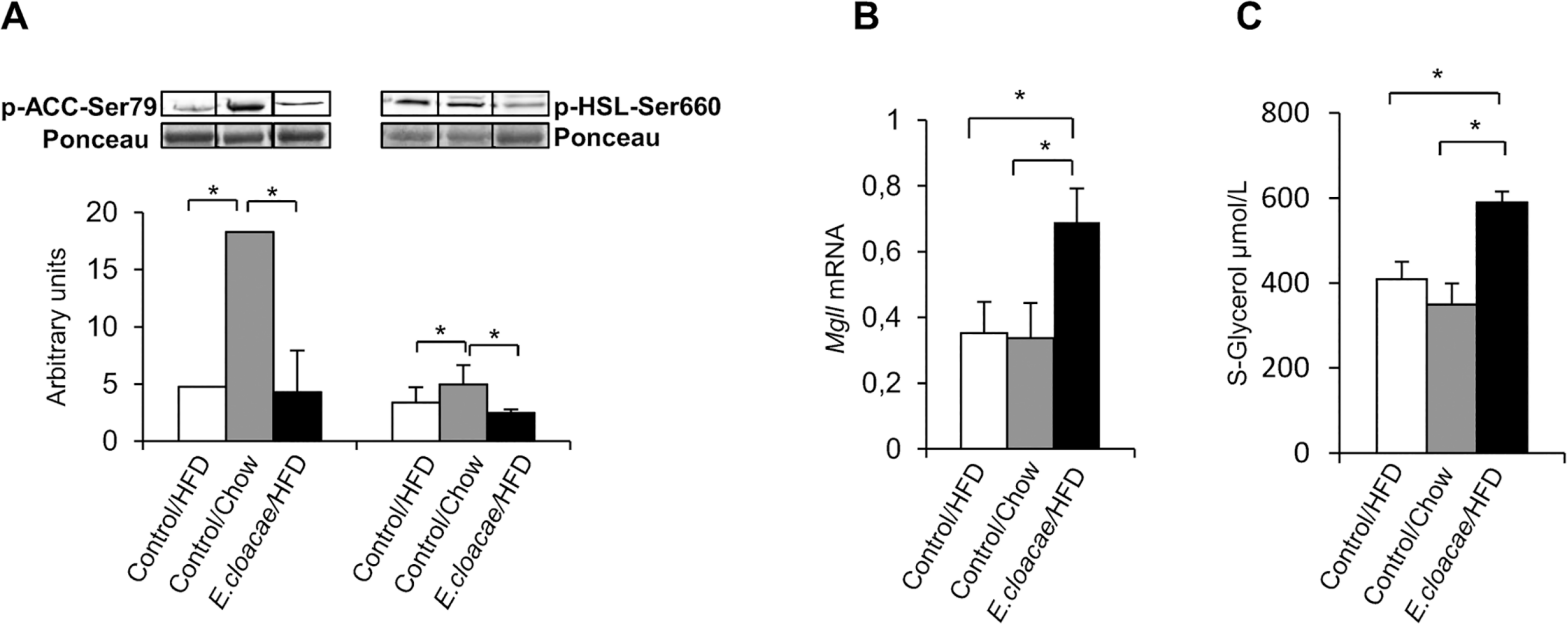
*E*. *cloacae* administration increases lipolysis in SAT. At necropsy: (A) ACC and HSL phosphorylation; (B) *Mgll* expression; (C) Serum glycerol levels. All data are presented as mean ± SD. n was 4-6/group when statistical outliers were excluded. The statistical significance was set to p<0.05 and the significant differences are presented with lines and * between the groups.

As an indication of enhanced fatty acid oxidation, the chow control group expressed more phosphorylated Acetyl-CoA carboxylase (ACC) compared to the HFD controls (p = 0.014, Fig 2A) and the *E*. *cloacae* treatment group (p = 0.01, Fig 2A). Surprisingly, however, chow controls had increased hormone sensitive lipase (HSL) phosphorylation compared to the HFD controls and the *E*. *cloacae* -treated mice (p = 0.01 and p = 0.021, respectively, Fig 2A). HSL induces lipolysis and has been argued to contribute to insulin resistance and NAFLD by releasing glycerol to the portal vein [24]. Monoacylglycerol lipase (MGLL) functions together with HSL to hydrolyze intracellular triglyceride stores in adipocytes and other cells to fatty acids and glycerol. *E*. *cloacae* treatment increased the *Mgll* expression compared to both HFD (p = 0.016) and chow control groups (p = 0.004, Fig 2B). Consequently, the *E*. *cloacae* treatment group had higher serum glycerol levels than the HFD and chow control mice (p = 0.029 and p = 0.016, respectively, Fig 2C).

**Fig 3:**
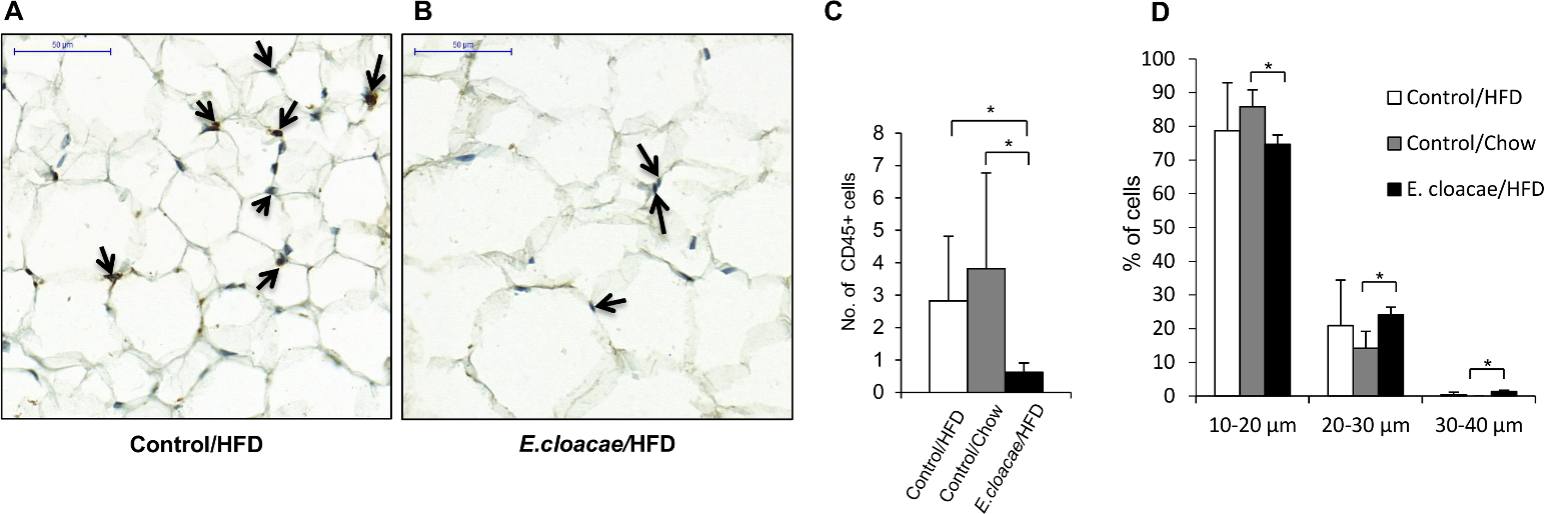
*E*.*cloacae* adminstration decreases the number of leukocytes and increases the size of adipocytes. (A) and (B) The histological images present the adipose tissues with CD45-staining indicated with arrows. (C) The relative numbers of CD45-positive cells as determined with manual cell counting from the stained tissues, represented as average cell numbers per field of view. (D) The percentages of adipocytes with diameters of 10–20 μm, 20–30 μm and 30–40 μm in each group. 500 cells were randomly selected from each sample. All data are presented as mean ± SD. n was 5-6/group. The statistical significance was set to p<0.05 and the significant differences are presented with lines and * between the groups.

Fig 3A and 3B show typical CD45 staining of the SAT of HFD control and *E*. *cloacae*-treated mice. *E*. *cloacae* -treated mice had significantly less CD45-positive cells in the SAT than the HFD and chow controls (p = 0.008 and p = 0.004, respectively, Fig 3C). Based on cell size counting from the H&E stained subcutaneous adipose tissue, *E cloacae*-treated mice had significantly higher percentage of hypertrophic (30–40 μM) adipocytes than the chow controls (p = 0.016, Fig 3D), while the difference between HFD controls and chow controls was not statistically significant.

### *E*. *cloacae* administration increases lipolysis, but also increases adiponectin expression in visceral adipose tissue

**Fig 4:**
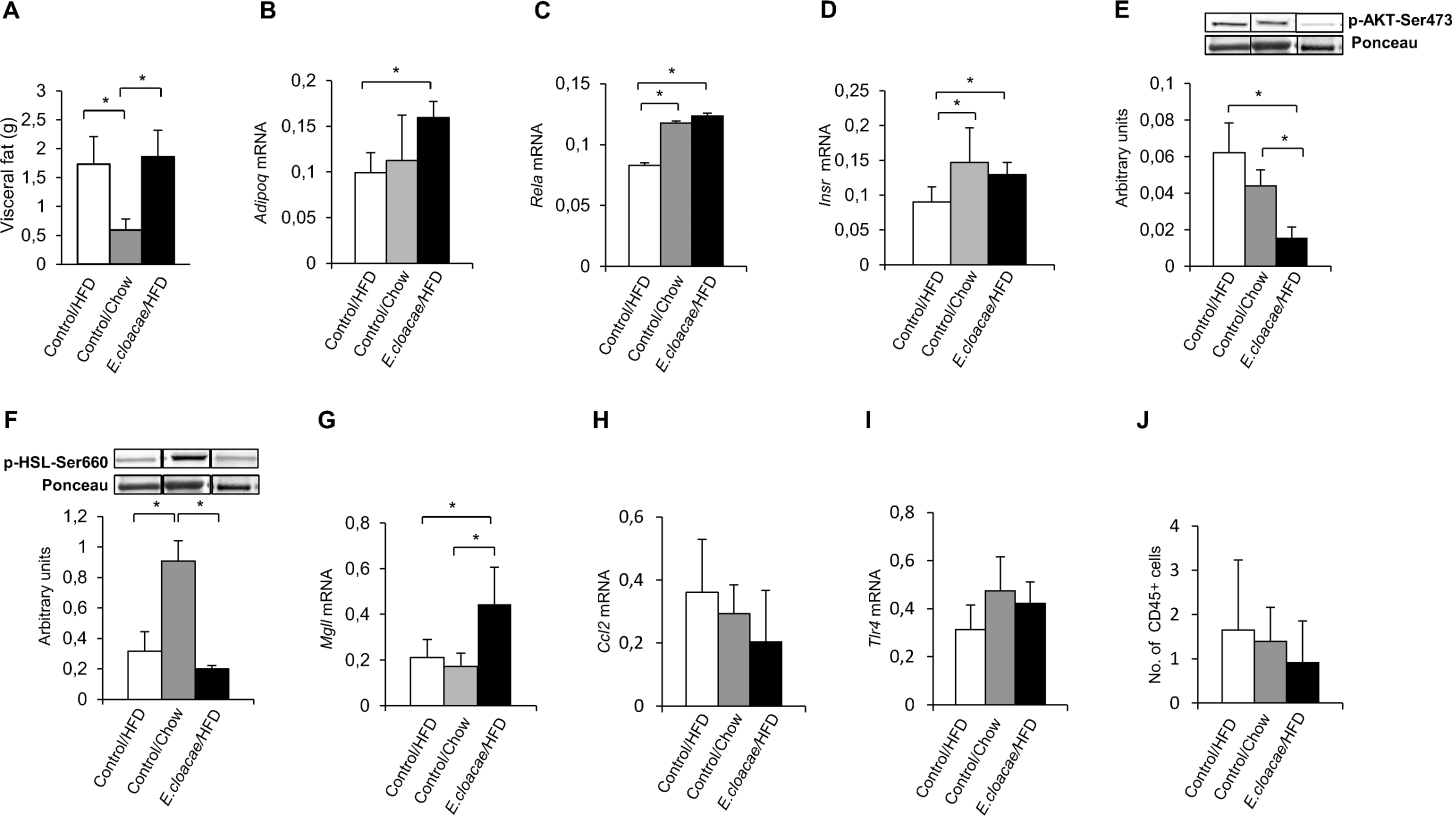
*E*. *cloacae* administration increases insulin resistance and lipolysis, but also increases adiponectin expression in VAT. At necropsy (A) visceral fat mass; (B) *Adipoq* expression; (C) *Rela* expression; (D) *Insr* expression; (E) AKT phosphorylation; (F) HSL phosphorylation; (G) *Mgll* expression; (H) *Ccl2* expression; (I) *Tlr4* expression; (J) The relative numbers of CD45-positive cells as determined with manual cell counting from the stained tissues, represented as average cell numbers per field of view. All data are presented as mean ± SD. n was 4-6/group when statistical outliers were excluded. The statistical significance was set to p<0.05 and the significant differences are presented with lines and * between the groups.

VAT drains to the portal vein, and therefore liver is directly exposed to cytokines and adipokines secreted by this tissue [25]. While the *E*. *cloacae* -treated mice differed only from the chow controls in the VAT mass (p = 0.004, Fig 4A), surprisingly, the expression of VAT-specific adipokine adiponectin that enhances hepatic fat oxidation [26], was increased in the *E*. *cloacae* -treated mice compared to the HFD controls (*Adipoq*, p = 0.029, Fig 4B). Both the *E*. *cloacae -*treated mice and chow controls expressed more Nuclear factor kappa B (NF-κB) p65 encoding gene (*Rela*) than the HFD controls (p = 0.016 and 0.004, respectively, Fig 4C). In addition, the *E*. *cloacae* treatment group and chow controls had higher expression levels of *Insr* than the HFD control group (p = 0.016 and 0.004, respectively, Fig 4D). However, the phosphorylation of insulin-sensitive Ser473 of AKT was decreased in the VAT of *E*. *cloacae* -treated mice compared to the HFD controls (p = 0.029, Fig 4E) and Ser660 of HSL compared to the chow controls (p = 0.029, Fig 4F). *E*. *cloacae* treatment increased the *Mgll* expression compared to chow control group (p = 0.008), and tended to increase the expression compared to the HFD controls (p = 0.063, Fig 4G). There were no differences in the *Ccl2* expression between the study groups (Fig 4H). Surprisingly, *Tlr4* on expression seemed to be highest in the chow controls, but the differences between the groups were not statistically significant (Fig 4I). Further, no differences were observed in the relative number of CD45-positive cells between the study groups (p>0.1 for all; Fig 4J).

### *E*. *cloacae* administration increases hepatic AST activity and triglyceride synthesizing *Dgat2* expression, without increasing triglyceride content

**Fig 5:**
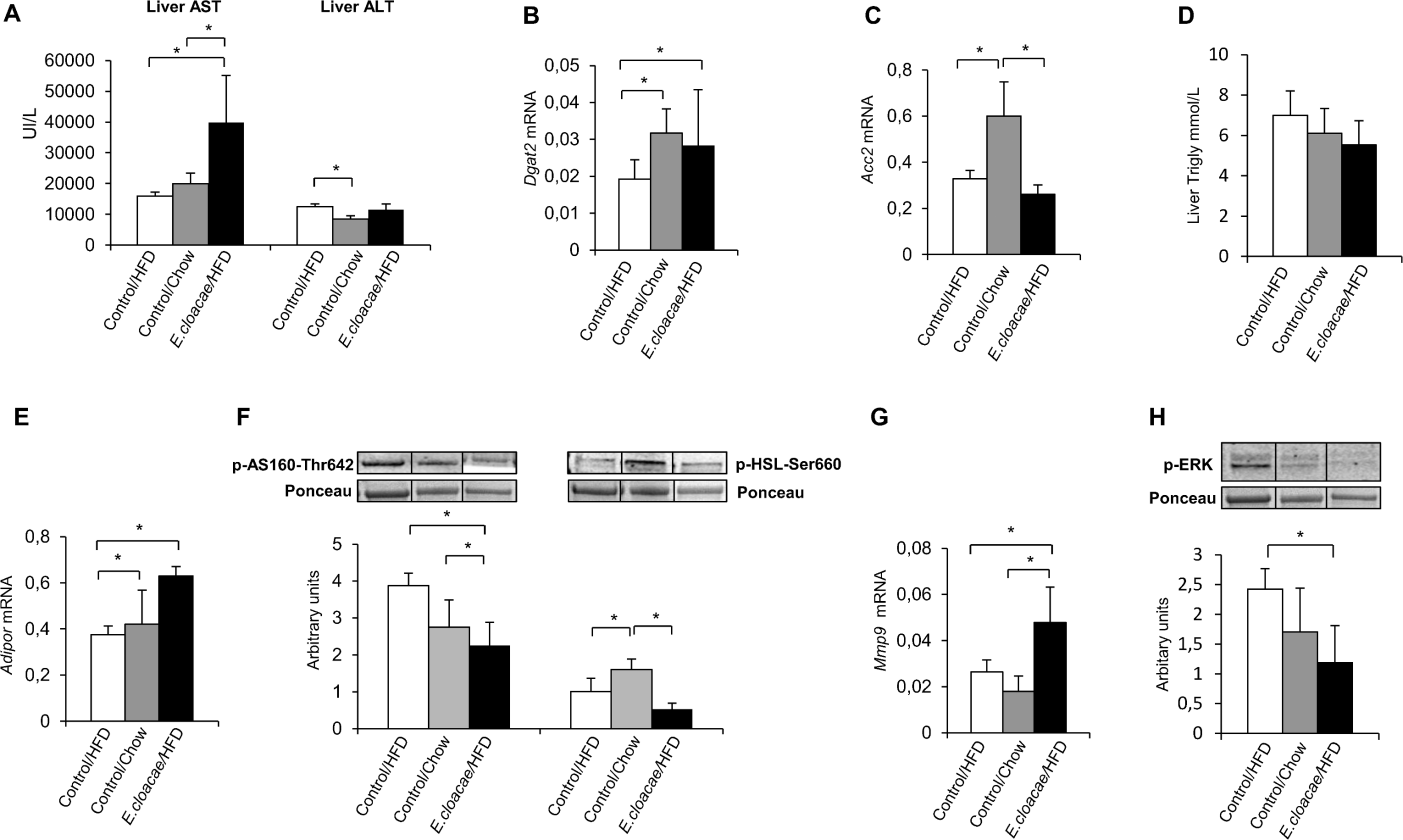
*E*. *cloacae* administration increases hepatic AST activity and triglyceride synthesizing *Dgat2* expression without increasing triglyceride content. At necropsy: (A) hepatic AST and ALT activity; (B) *Dgat2* expression; (C) *Acc2* expression; (D) triglyceride content; (E) *Adipor* expression; (F) AS160 and HSL phosphorylation; (G) *Mmp9* expression; (H) ERK phosphorylation. All data are presented as mean ± SD. n was 4-6/group when statistical outliers were excluded. The statistical significance was set to p<0.05 and the significant differences are presented with lines and * between the groups.

The mice treated with *E*. *cloacae* had significantly higher hepatic AST activity than the HFD and chow controls (p = 0.016 and p = 0.004, respectively, Fig 5A), and the chow controls had lower ALT than the HFD controls (p = 0.01, Fig 5A). The *E*. *cloacae* -treated mice expressed more triglyceride-synthesizing *Dgat2* than the HFD controls (p = 0.032, Fig 5B), while the chow controls expressed more *Acc2* and *Dgat2* than the HFD controls (p = 0.016 and 0.009, respectively, Fig 5C). However, the groups did not differ from each other in the hepatic triglyceride content (Fig 5D), which may be due to increased fat metabolism-enhancing adiponectin receptor, *Adipor*, expression in the *E*. *cloacae* treatment group compared to the controls (p = 0.016 for both, Fig 5E).

**Fig 6:**
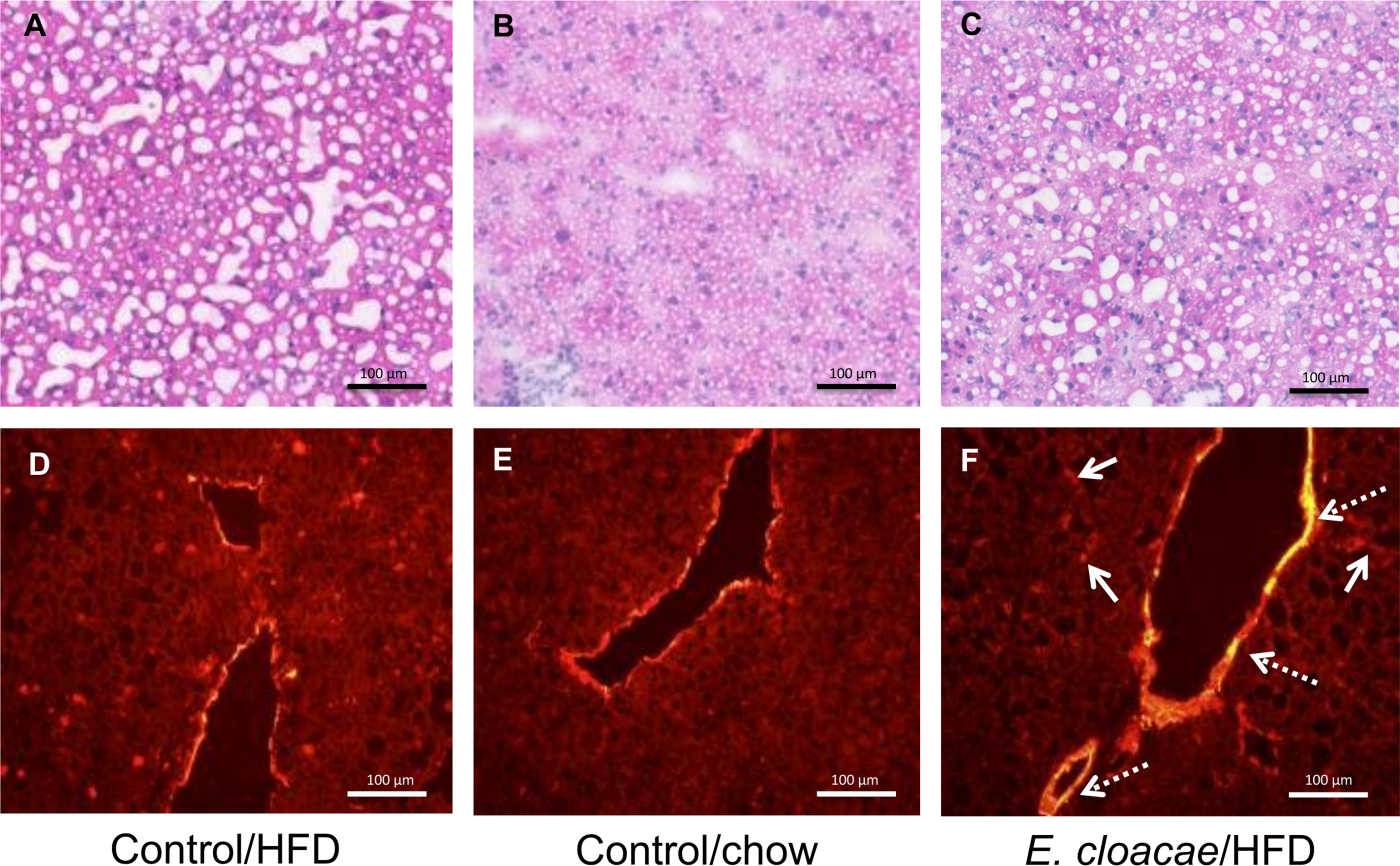
Fibrosis is increased in the liver of *E*. *cloacae* -treated mice. (A), (B), and (C) H&E staining; (D), (E) and (F) overexposed anti -smooth muscle actin stainings of the frozen liver tissues. H&E staining shows more ballooning and an increase in lipid droplets in HFD controls and *E*. *cloacae* -treated mice compared to chow controls. In SMA staining, an increase in *E*. *cloacae* -treated mice compared to chow controls and HFD controls can be seen both in blood vessels (discontinuous arrows) and sites outside the vessels (continuous arrows).

*E*. *cloacae* -treated mice had decreased AS160 phosphorylation levels compared to the HFD and chow controls (p = 0.021 and p = 0.032, respectively, Fig 5F), potentially affecting the glucose incorporation into glycogen in liver [27]. In addition, the *E*. *cloacae* treatment increased Matrix metalloproteinase-9 (*Mmp9)* expression compared to the HFD and chow controls (p = 0.049 and 0.008, respectively, Fig 5G), which can be an indication of extracellular matrix remodeling [28]. Further, *E*. *cloacae* -treated mice had significantly decreased ERK phosphorylation levels compared to the HFD controls (p = 0.029, Fig 5H). Liver from HFD control, chow control and *E*. *cloacae*-treated mice was stained for SMA to visualize fibrosis and with H&E for overall morphology and lipid droplet visualization. Typical stainings are presented in Fig 6. H&E-stained liver sections showed significantly lower hepatic fat content and ballooning in the chow controls compared to the HFD controls and the *E*. *cloacae* -treated mice (Fig 5A–5C). Based on the SMA staining, *E*. *cloacae* -treated mice tended to express more SMA than the HFD and chow control mice both in and outside of large blood vessels (Fig 5D–5F), which may indicate fibrosis.

### *E*. *cloacae* treatment increased the intestinal Toll like receptor 5 gene expression

**Fig 7:**
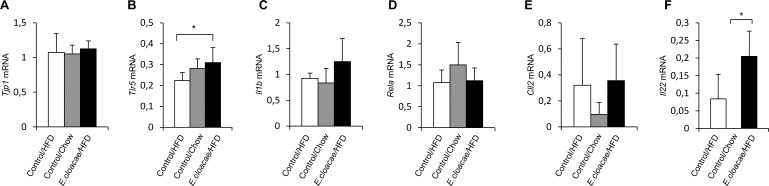
*E*. *cloacae* administration increases TLR5 and IL-22 gene expression. At necropsy: (A) *Tjp1* expsession; (B) *Tlr5* expression; (C) *Il1b* expression; (D) *Rela* expression; (E) *Ccl2* expression; (F) *Il22* expression. All data are presented as mean ± SD. n was 4-6/group when statistical outliers were excluded. The statistical significance was set to p<0.05 and the significant differences are presented with lines and * between the groups.

Compared to the HFD control group, the intragastric delivery of *E*. *cloacae* increased the expression of Toll-like receptor 5-encoding gene *Tlr5* (p = 0.032, Fig 7A). The expression of genes encoding tight junction protein 1 (*Tjp1*), NF-κB (*Rela*) or interleukin 1β (*Il1b*) did not differ between the groups (p>0.1 for all, Fig 7B–7D). Further, no statistically significant differences were observed in the intestinal expression of *Ccl2* between the study groups (Fig 7E). Interestingly, however, the expression of *Il22* was significantly increased in the *E*. *cloacae* -treated mice compared to the chow controls (p = 0.009, Fig 7F). The difference between the *E*. *cloacae* treatment group and the HFD controls was not statistically significant (p = 0.063; Fig 7F).

## Discussion

Human studies have reported an increased abundance of *Enterobacteriaceae* in obesity [14,29]. These bacteria have also been related to defective hepatic functions, as the abundance of *Proteobacteria* phyla including the *Enterobacteriaceae* family is suggested to gradually increase from healthy to obese subjects, and further to subjects with NASH [30]. In addition, *Enterobacteriaceae* family has been reported to increase in patients with liver cirrhosis [31]. In this study, we showed that *E*. *cloacae* ATCC® 13047™ treatment combined with HFD caused liver damage and seemed to increase adipose tissue hypertrophy in mice. In agreement, Fei & Zhao reported that *E*. *cloacae* B29, a close phylogenetic relative to *E*. *cloacae* ATCC® 13047™ isolated from a morbidly obese volunteer, induced obesity and liver injure in mice on HFD [14].

Adipose tissue expands when energy is harvested from HFD or because of *de novo* lipogenesis. In obesity-associated insulin resistance, however, the absence of adequate insulin signaling permits unregulated adipose tissue lipolysis due to lipid overload [32]. In lipolysis, three lipases act in sequence releasing one free fatty acid in each step: adipose triglyceride lipase (ATGL) converts triglycerides to diacylglycerols that are hydrolyzed to monoglycerides by HSL. Finally, MGLL cleaves monoglycerides into free fatty acids and glycerol that are released to the circulation [33]. Our results suggest that *E*. *cloacae* treatment might have caused partial insulin resistance, as *Insr* expression was reduced and subsequently lipolysis was increased (seen as increased *Mgll* expression). This was reflected in higher serum levels of glycerol compared to the HFD controls. In addition to decreased SAT *Insr*, hepatic insulin-sensitive ERK phosphorylation was decreased in *E*. *cloacae* -treated mice, also discreetly supporting decreased insulin sensitivity. However, no insulin tolerance tests were performed to confirm that these findings are due to insulin resistance. Further, the fasting of the animals may have affected the results, as fasting may have had a distinct effect on the mice on chow diet compared to the mice on HFD.

Impaired regulation of adipose tissue lipolysis can result in adipocyte hypertrophy, which is suggested to contribute to the ER stress and inflammatory cell infiltration, and to exacerbate insulin resistance [34,35]. Conversely, ER stress can be alleviated by XBP1 that regulates ER-resident chaperone genes in the unfold protein response, subsequently reducing the production of inflammation-inducing reactive oxygen species [36,37]. However, contradictory findings suggest that XBP-1, which has glucose-lowering and insulin-sensitizing effect in obesity, induces, in concert with NF-κB, the transcription of genes encoding inflammatory cytokines such as interleukin 1β [38]. We found that *E*. *cloacae*-treated mice had more hypertrophic adipocytes compared to the chow controls, while the difference between the HFD controls and the chow controls was not statistically significant. This indicates that *E*.*cloacae* treatment might have induced adipose cell hypertrophy. Accordingly, the expression of *Ccl2* was significantly elevated in the *E*. *cloacae* -treated mice. *Ccl2* encodes for MCP-1, a pro-inflammatory chemokine that is suggested to stimulate the recruitment of macrophages and dendritic cells, and to further increase the expression of cytokines that exacerbate inflammation-induced insulin resistance [39]. Yet, surprisingly, the *E*. *cloacae* -treated mice had less CD45-positive leukocytes in their SAT compared to the HFD controls. The specific number of macrophages was, however, not analyzed. Moreover, we did not observe induction in *Rela* or *Il1b* (data not shown) in SAT of *E*. *cloacae*-treated mice, while the expression of *Xbp1* and *Tlr4* actually were reduced. Our findings would therefore suggest that the lower expression of *Xbp1* and *Tlr4* may contribute to the lower expression of *Rela* and *Il1b*. Notwithstanding, it is possible that the inflammation takes place later in the *E*. *cloacae* -treated mice, when the hypertrophy due to hypoxia and oxidative stress leads to pyroptotic cell death [35]. Nevertheless, recent studies suggest that, in contrast to systemic inflammation, local inflammation in adipose tissue may be protective against insulin resistance [40,41]. Local proinflammatory response seem to permit healthy adipose tissue expansion by increasing adipogenesis instead of hypertrophy, which further impedes ectopic lipid accumulation in other tissues and thus metabolic derangements [42]. This theory would be in agreement with the presence of hypertrophic adipocytes and the lack of inflammation detected in *E*. *cloacae*-treated mice.

In VAT, by contrast, increased *Rela* expression was observed, suggesting the presence of inflammation. This finding is consistent with Fei & Zhao reporting increased inflammation in the epididymal fat in response to *E*. *cloacae* B29 [14]. The increased inflammation in *E*. *cloacae* -treated mice was accompanied with lower AKT phosphorylation and higher expression of *Mgll* that have possibly contributed to glycerol release. Nevertheless, surprisingly, no differences were observed in the number of CD45-positive cells or in the expression of *Ccl2* or *Tlr4* between the study groups. Further, *Insr* and adiponectin-encoding *Adipoq* were increased in the VAT of *E*. *cloacae* -treated mice. Adiponectin has several insulin-sensitizing effects at the whole organism level [26]. A recent study showed that LPS from Gram-negative bacteria inhibited the secretion of adiponectin [43]. Contrarily, in this study, *E*. *cloacae* increased adiponectin expression, which is also opposite to the reported effects of *E*. *cloacae* B29 [14]. It remains to be determined whether the different findings are due to strain-specific effects. On the other hand, one possible reason for these partly inconsistent results is the relatively infrequent *E*. *cloacae* inoculations during the treatment period; more explicit results may have been obtained with more frequent administrations and/or higher dosage. Further, as fecal samples were not analyzed, it cannot be confirmed that the colonization of the bacterium was successful in all *E*. *cloacae* -treated mice, even though it has been previously shown that intragastric *E*. *cloacae* administration leads to long-lasting gut colonization [14]. Further, the possible presence of *E*. *cloacae* or LPS in the circulation or in the tissues of the mice was not analyzed in this study. The intestinal *Tjp1* expression results, alongside with low LPS-recognizing TLR4 gene expression in the adipose tissue, might suggest that no remarkable leakage from the gut occurred, but this cannot be fully confirmed based on these results.

As a consequence of adipose tissue lipolysis, glycerol and fatty acids are released to the circulation. The majority of fatty acids are metabolized to acetyl-coA for generation of energy in all oxidative tissues [32]. Glycerol will be taken up by liver, where it serves as a substrate for triglyceride synthesis by DGAT2, but is also frequently utilized as gluconeogenic substrate [44]. Despite the higher serum glycerol and higher expression levels of *Dgat2*, the *E*. *cloacae* -treated mice did not differ from the HFD controls on the hepatic lipid content. This may be attributed, at least partly, to the increased adiponectin and adiponectin receptor expression in response to *E*. *cloacae*, as adiponectin signaling enhances oxidative lipid metabolism in liver [45]. On the other hand, *E*. *cloacae* treatment resulted in lower phosphorylation levels of AS160, which may have affected the glucose incorporation into glycogen [27], thus suggesting that more glucose could have been available for hepatic *de novo* lipogenesis.

*E*. *cloacae* treatment significantly increased AST and consequently AST/ALT ratio to over 2:1, which is an indication of liver fibrosis or cirrhosis [46]. Fei & Zhao reported that *E*. *cloacae* B29 also caused liver damage [14]. The state of liver fibrosis is supported by the increased *Mmp9* expression levels in *E*. *cloacae*-treated mice. In steatotic and fibrotic liver, *Mmp9* has been shown to increase extracellular matrix remodeling to promote leukocyte infiltration and angiogenesis [47]. In accordance, SMA seemed to increase in the liver of *E*. *cloacae* -treated mice compared to both control groups. Increased expression of SMA is related to the formation of vasculature and activation of hepatic stellate cells that drive liver fibrosis and the progression of hepatic steatosis to NASH [48].

Liver damage has been associated with increased intestinal inflammation and permeability [49]. Recently, Yan *et al*. reported increased colonic inflammatory conditions as a response to *E*. *cloacae* B29 treatment on germ-free mice on HFD [19]. Nonetheless, in our study, no differences in the mRNA levels of several inflammatory markers or tight junction protein 1, describing the integrity of the gut epithelium, were found in the *E*. *cloacae* -treated mice compared to the control groups. Interestingly, however, flagellin-recognizing *Tlr5* expression was increased in the *E*. *cloacae* treatment group. Recent studies have linked TLR5 and flagellin to the intestinal inflammation [50]. In the future, it should be studied whether the flagellated *E*. *cloacae* can activate TLR5. In addition, the intestinal *Il22* expression was significantly increased in the *E*. *cloacae* treatment group compared to the chow controls. IL-22 has an important role in maintaining the intestinal epithelial barrier and preventing epithelial damage induced by bacteria and inflammation [51]. These results thus suggest that *E*. *cloacae* administration, accompanied by HFD, has activated the intestinal defense mechanisms. However, as the gut microbiota composition was not analyzed, it cannot be concluded whether this activation was a direct consequence of *E*. *cloacae* colonization or arose from more complex chances in gut microbiota. Further, only female mice were analyzed in this study. Previous studies have shown that both gut microbiota composition and immunity are, to some extent, sex-dependent [52,53], and thus the findings presented in this study might not be seen in male mice.

### Conclusions

**Fig 8:**
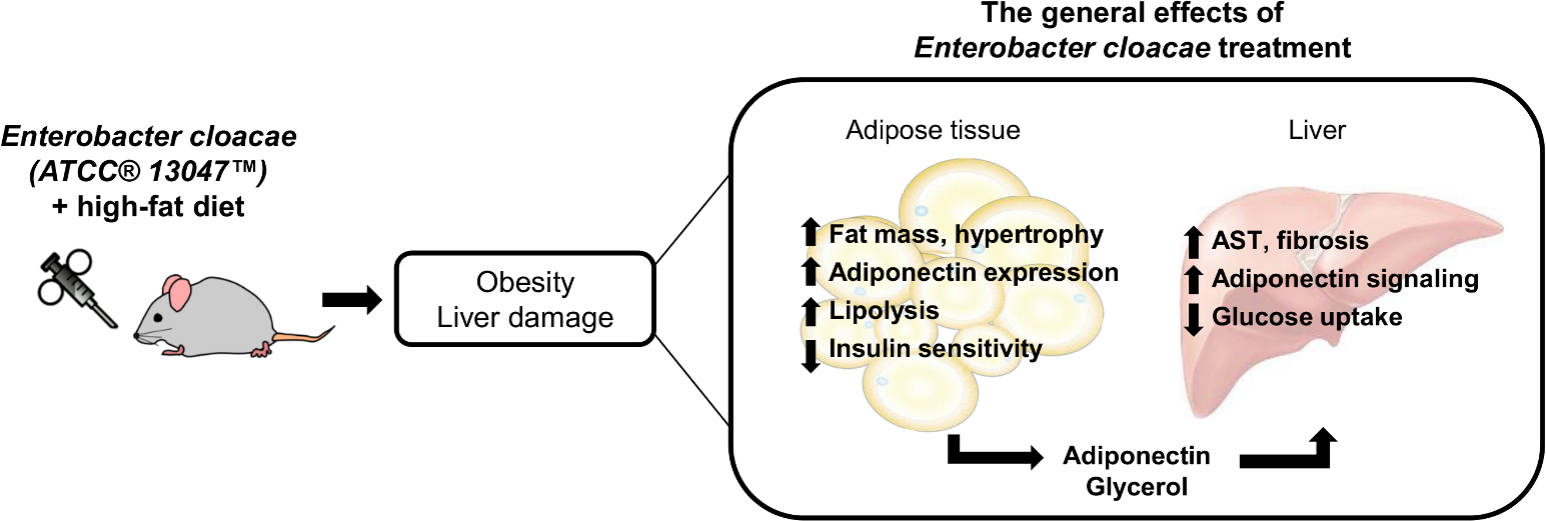
Summary of the main findings. *E*. *cloacae* administration increased adipose tissue hypertrophy and hepatic damage in the HFD fed mice. The subcutaneous adipose tissue of the *E*. *cloacae* -treated mice appeared to be partly insulin resistant, and the increased lipolysis and adipocyte hypertrophy led to increased glycerol release. Liver fat accumulation did not increase in response to the *E*. *cloacae* treatment, but AST activity measurements and histology revealed hepatic damage in the *E*. *cloacae* -treated mice.

In conclusion, we showed that the *E*. *cloacae* treatment increased the SAT mass in mice, and that the SAT of *E*. *cloacae* -treated mice might have been insulin resistant. In addition, the treatment increased lipolysis and adipocyte hypertrophy that led to increased glycerol release. Nevertheless, likely due to increased adiponectin signaling, fat did not accumulate in the liver. Importantly, however, AST activity measurements and histology showed hepatic damage in response to the *E*. *cloacae* treatment. The main findings of this study are summarized in Fig 8.

## Supporting information

S1 Table PCR primers used in this study. (PDF)
