# Supplementary material for: Enterobacter cloacae administration induces hepatic damage and subcutaneous fat accumulation in high-fat diet fed mice
Source: PLoS One. 2018 May 30;13(5):e0198262. doi: 10.1371/journal.pone.0198262 (PMC5976205; doi:10.1371/journal.pone.0198262)
Supplement: S1 Table — (PDF) [file pone.0198262.s001.pdf]

S1 Table. PCR primers used in this study.

**Primer name**	**Primer sequence 5’ > 3’**
mDgat2 fwd	CGCAGCGAAAACAAGAATAA
mDgat2 rev	GAAGATGTCTTGGAGGGCTG
mAdipoq fwd	CCTGGAGAGAAGGGAGAGAAA
mAdipoq rev	CGAATGGGTACATTGGGAAC
mMmp9 fwd	AGACGACATAGACGGCATCC
mMmp9 rev	CTGTCGGCTGTGGTTCAGT
mAcc2 fwd	GAGGCTGCATTGAACACAAG
mAcc2 rev	TGTTCTCGGCCTCTCTTCAC
mInsr fwd	CAGCCGGATGGGCCAATGGGA
mInsr rev	CTCGTCCGGCACGTACACAGAA
mXbp1 fwd	TGCTGAGTCCGCAGCAGGTG
mXbp1 rev	CTGATGAGGTCCCCACTGACAGA
mIl1b fwd	TGTGAAATGCCACCTTTTGA
mIl1b rev	GGTCAAAGGTTTGGAAGCAG
mAdipor fwd	GGAGTGTTCGTGGGCTTAGG
mAdipor rev	GCAGCTCCGGTGATATAGAGG
mACTB fw	GGCTGTATTCCCCTCCATCG
mACTB rev	CCAGTTGGTAACAATGCCATGT
mRela fwd	TTTCGATTCCGCTATGTGTG
mRela rev	GAACGATAACCTTTGCAGGC
mTlr5 fwd	AAGTTCCGGGGAATCTGTTT
mTlr5 rev	GCATAGCCTGAGCCTGTTTC
mTjp1 fwd	GAGCGGGCTACCTTACTGAAC
mTjp1 rev	GTCATCTCTTTCCGAGGCATTAG
mMgll fwd	AAAGTTTGTCGGAGAATCGG
mMgll rev	TTTTCCAGAACACACCCCTG
mTlr4 fwd	CAAGAACATAGATCTGAGCTTCAACCCGCT
mTlr4 rev	GTCCAATAGGGAAGCTTTCTAGAG
mCll2 fwd	AGGTGTCCCAAAGAAGCTGTA
mCll2 rev	ATGTCTGGACCCATTCCTTCT
